# The Effects of Hemodialysis and Peritoneal Dialysis on the Gut Microbiota of End-Stage Renal Disease Patients, and the Relationship Between Gut Microbiota and Patient Prognoses

**DOI:** 10.3389/fcimb.2021.579386

**Published:** 2021-03-23

**Authors:** Dan Luo, Wenbo Zhao, Zhiming Lin, Jianhao Wu, Hongchun Lin, Yongjie Li, Jun Song, Jun Zhang, Hui Peng

**Affiliations:** ^1^ Department of Nephrology, The Third Affiliated Hospital of Sun Yat-sen University, Guangzhou, China; ^2^ Department of Nephrology, The First Affiliated Hospital of Sun Yat-sen University, Guangzhou, China; ^3^ Department of Rheumatology and Immunology, The Third Affiliated Hospital of Sun Yat-sen University, Guangzhou, China

**Keywords:** gut microbiota, dialysis, end-stage renal disease, 16S rRNA sequencing, prognosis, bioinformatics

## Abstract

Gut microbiota alterations occur in end-stage renal disease (ESRD) patients with or without dialysis. However, it remains unclear whether changes in gut microbiota of dialysis ESRD patients result from dialysis or ESRD, or both. Similarly, there is a dearth of information on the relationship between gut microbiota and ESRD prognoses. We collected fecal samples and tracked clinical outcomes from 73 ESRD patients, including 33 pre-dialysis ESRD patients, 19 peritoneal dialysis (PD) patients, and 21 hemodialysis (HD) patients. 16S rRNA sequencing and bioinformatics tools were used to analyze the gut microbiota of ESRD patients and healthy controls. Gut microbiota diversity was different before and after dialysis. Bacteroidetes were significantly deceased in HD patients. Twelve bacterial genera exhibited statistically significant differences, due to dialysis (all *P* < 0.05, FDR corrected). HD reversed abnormal changes in *Oscillospira* and *SMB53* in pre-dialysis patients. Functional predictions of microbial communities showed that PD and HD altered signal transduction and metabolic pathways in ESRD patients. Furthermore, *Bacteroides* and *Phascolarctobacterium* were associated with cardiovascular mortality. *Dorea*, *Clostridium*, and *SMB53* were related to peritonitis in PD patients. This study not only demonstrated differences in gut microbiota between pre-dialysis and dialysis ESRD patients, but also firstly proposed gut bacteria may exert an impact on patient prognosis.

## Introduction

Chronic kidney disease (CKD) leads to a gradual loss of kidney function, eventually developing to end-stage renal disease (ESRD) (Webster et al., 2017). Renal replacement therapies (RRT) are required for ESRD patient survival, including hemodialysis (HD), peritoneal dialysis (PD), or kidney transplantation. It was predicated that worldwide RRT would more than double to 5.439 million people by 2030, with the greatest growth in Asia (Liyanage et al., 2015). Therefore, these data show a pressing need to understand the impact of dialysis on the body and improve treatments to reduce disease complications and mortality.

There is a growing acceptance that gut microbiota and its products affect CKD pathogenesis and development (Yang et al., 2018; Meijers et al., 2019; Hu et al., 2020a). Several factors such as toxin accumulation, chronic inflammatory status, drug (corticosteroids, immunosuppressive agents, antibiotics) treatments, and dietary restrictions could affect gut microbiota (Simoes-Silva et al., 2018). HD and PD both remove excess water from the body to reduce volume load, remove metabolic wastes (such as creatinine, urea nitrogen, and other small molecule toxins) and maintain electrolyte and acid-base balance (Velasquez et al., 2018). These factors suggest that dialysis may improve gut dysbiosis. But due to catheter intervention or adverse complications during dialysis, these factors may also disrupt gut microbiota composition. However, few studies have documented the effects of HD and PD on gut microbiota in ESRD patients. Recent studies have found differences in gut microbiota composition and function between dialysis ESRD patients and healthy controls (Crespo-Salgado et al., 2016; Stadlbauer et al., 2017; Gao et al., 2020). However, these results were not consistent, more importantly, these studies did not compare dialysis ESRD patients with pre-dialysis ESRD patients, the findings of the changes in the gut microbiota of dialysis ESRD patients can be a consequence of dialysis or ESRD itself (or both). In response to this problem, it is vital to confirm the influence of HD and PD on gut microbiota in ESRD patients.

Cardiovascular (CV) disease and infections are common complications in ESRD patients, with CV diseases being the main cause of mortality in such patients (Webster et al., 2017; Cozzolino et al., 2018). Bacterial peritonitis remains the main infectious complication in PD patients (Ballinger et al., 2014). It has been shown that CV disease and infections are closely associated with gut dysbiosis (Velasquez et al., 2018; Onal et al., 2019). A study revealed a key role of the peritoneal microbiota to promote infection occurrence or progression in ESRD patients, especially PD patients (Simoes-Silva et al., 2020). However, the relationship between the gut microbiota and peritonitis is still poorly understood. In recent years, the relationship between PD patients and the gut microbiota has been investigated to determine possible therapeutic targets (prebiotics and probiotics, such as p-Inulin), to improve patient quality of life and improved survival for ESRD patients (Gao et al., 2020).

Herein, we explore the altered composition and function of gut microbiota in ESRD patients undergoing dialysis, using high-throughput sequencing of the 16S rDNA V3–V4 variable region. We also analyzed correlations between changes in gut microbiota and clinical parameters. More importantly, we investigated the relationship between gut microbiota and the clinical outcomes of ESRD patients.

## Materials and Methods

### Study Population

This study was conducted at The Third Affiliated Hospital of Sun Yat-sen University, and was approved by the ethics review board of The Third Affiliated Hospital of Sun Yat-sen University [(2018) 02-320-01]. All eligible subjects were ≥ 18 years old and provided written informed consent. In total, 92 subjects were included and divided into four groups: (1) 19 healthy volunteers with normal renal function (healthy control group), (2) 33 pre-dialysis ESRD patients (ND group), (3) 19 ESRD patients with continuous regular PD for > 6 months (PD group), (4) and 21 ESRD patients with continuous regular HD for > 6 months (HD group). ESRD diagnostic criteria included: glomerular filtration rate calculated using the CKD-EPI formula, the glomerular filtration rate was < 15 mL/(min 1.73 m^2^) (National Kidney, 2002). Exclusion criteria were: patients with secondary nephropathy, malignancy, pregnancy, acute or chronic infections, patients treated with antibiotics, probiotics, prebiotics, glucocorticoids, immunosuppressive drugs during the three months before fecal sample collection, those diagnosed with end-stage liver disease or chronic gut-related diseases, those unable to eat by themselves, or using enteral and external nutrition intervention. Clinical and biochemical data were collected from medical records.

During follow-up (August 2018–May 2020), we tracked patient clinical outcomes, including death and peritonitis. Cardiovascular (CV) disease is the group of disorders of the heart and blood vessels, including hypertension, coronary heart disease, cerebrovascular disease, peripheral vascular disease, heart failure, rheumatic heart disease, congenital heart disease, and cardiomyopathy (World Health Organisation). CV death was defined as death due to a CV event, including congestive heart failure, coronary heart disease, fatal stroke, cardiac arrhythmia, sudden cardiac arrest, cerebrovascular disease, and peripheral vascular disease (Zhong et al., 2020).

### Sample Collection and 16S Sequencing

Fecal samples were collected between August 2018 and September 2019. Samples were frozen within 24 h of receipt. Microbial DNA was extracted from samples using the QIAamp Fast DNA Stool Mini Kit (QIAGEN, Hilden, Germany), according to manufacturer’s instructions. All processed human fecal genomic DNA (gDNA) was PCR amplified using V3F (5’-ACTCCTACGGGAGGCAGCA-3’) and V4R (5’-GGACTACHVGGGTWTCTAAT-3’) primers (Invitrogen Carlsbad, CA, USA) to amplify the 16S rRNA gene, specifically the V3–V4 variable region. The protocol for amplification includes:95°C for 5 min, 30 cycles, 95°C for 30 s, 53°C for 30 s, and 72°C for 30 s. The final elongation was maintained at 72°C for 8 min. Each 20-μL PCR reaction contained 50 ng of template DNA, 10 μL of 2x Premix Taq (TaKaRa Biotechnology Co., Dalian, China), 0.4 μL of 10 μmol/L reverse and forward primer, and double-distilled water. PCR products were sequenced using the Illumina Hiseq 2500 (Illumina, San Diego, CA, USA). This platform uses a two-terminal sequencing pin-end (PE) method, with each sequence generating 250 (Hiseq) reads from 5’ and 3’ ends, respectively. Due to a certain proportion of sequencing errors in the original PE reads obtained by sequencing, the original data were cut and filtered before analysis, to remove low-quality reads to generate valid clean reads. Reads were spliced into tags by the overlapping relationship between PE reads, and further filtered to generate clean reads. Tags were clustered into operational taxonomic units (OTUs) for a given similarity and annotated to derive community composition information from each sample. For the prognosis analysis for CV, The OTU abundance analysis was based on ESRD patients; for the peritonitis in PD patients, the OTU abundance analysis was based on PD patients.

### Bioinformatics and Statistical Analysis

We used the MicrobiomeAnalyst website (https://www.microbiomeanalyst.ca) to conduct statistical analysis and data visualization (Chong et al., 2020). Chao1and Shannon indices were analyzed using Kruskal-Wallis test. In PCoA (Principal Co-Ordinates Analysis), we calculate the distance matrix using the Bray-Curtis dissimilarity method, which uses abundance data and calculates differences in feature abundance. The statistical method, permutational MANOVA (PERMANOVA) (also called Adonis), which is sensitive for multivariate dispersions, tests whether the centroids of all groups are equivalent. The approach uses distances (or dissimilarities) between samples of the same group, and compares them to distances between groups. R-squared represents the degree of explanation of the differences between samples by different groups. The larger the R-squared, the higher the degree of explanation of the differences of the group. When the P value is less than 0.05, the reliability of this test is high. The method of Analysis of similarities (ANOSIM) is also used to verify whether the differences between groups are significantly greater than the differences within the group, thus determining whether the grouping is meaningful. P value can judge whether the comparison between groups and within groups is significant or not. R value gives the degree of difference between and within groups. The actual range of R value is (−1, 1), but generally between (0, 1), R>0, indicating that there is a difference between groups; R< 0, indicating that the difference within groups is greater than the difference between groups. We used SparCC to calculate correlation networks. When compared with Pearson correlations and the hierarchical correlations of Spearman and Kendall, these simple methods often fail to assess microbiome compositional data and identify spurious correlations. SparCC uses logarithmic ratio transformations and performs multiple iterations to identify taxon pairs that are context-dependent outliers, fully assuming sparse correlation networks.

Phylogenetic investigation of communities by reconstruction of unobserved states (PICRUSt) analysis was used to predict metagenome function by annotating OTUs from the Greengenes database, as previously described (Langille et al., 2013). We translated species annotation information into gene identifiers on this database for analysis on the website (http://huttenhower.sph.harvard.edu/galaxy/). The results from the website which provided the OTU number of each sample in the Kyoto Encyclopedia of Genes and Genomes (KEGG) pathways were analyzed statistically using SPSS.

All other statistical analyses were performed using SPSS version 23.0 (SPSS Inc., Chicago, Illinois, USA) and GraphPad Prism version 7.00 for Windows (GraphPad Software, California, USA). We used the mean [± standard deviation (SD)] to express normally distributed data, and the median [Interquartile range (IQR)] to express skewed distribution data. A Chi-square test was used for the comparison of categorical data. When comparing normally distributed data with homogeneous variances, three or more groups were compared by ANOVA, using Bonferroni analysis. For parameters not normally distributed, non-parametric tests were used (Mann Whitney U test, or Kruskal-Wallis). Significant differences in relative abundances of genus among groups were corrected by Benn-Hochberg False discovery rate (FDR). All statistical tests were 2-sided, and *P*-values < 0.05 were considered statistically significant. The Spearman and Kendall correlation coefficient was calculated to estimate linear correlations between variables.

## Results

### Patient Clinical Characteristics

This study included 73 pre-dialysis and dialysis ESRD patients, and 19 healthy controls. The baseline characteristics of the ND, PD, HD, and control groups are shown (**Table 1**). We observed no differences in age, sex, body mass index (BMI), alanine amiotransferase (ALT), total cholesterol (TC), triglycerides (TG), low-density lipoprotein (LDL), high-density lipoprotein (HDL), white cells, and neutrophils amongst the four groups. As expected, ESRD patients showed impaired renal function and anemia symptoms, including significantly elevated blood urea nitrogen (BUN) and creatinine, and reduced hemoglobin (HB) and albumin (ALB) levels (**Table 1**). Other clinical and biochemical patient characteristics are shown (**Table 2**). Additionally, when compared with the ND group, both PD and HD groups had lower serum BUN levels. HD patients exhibited higher serum creatinine, ALB, HB, ferritin, parathyroid hormone (PTH), potassium, zinc, and magnesium levels, however, total iron-binding capacity (TIBC) and serum chloride levels were lower in HD patients. PD patients had lower homocysteine (HCY) and serum magnesium, and higher serum bicarbonate (HCO_3_) levels (**Tables 1** and **2**).

**Table 1:** The clinical characteristics of the four groups.

	ND group	PD group	HD group	CTL group	*P*1 value	*P*2 value
**Number**	33	19	21	19		
**Female/male**	18/15	8/11	9/12	9/10	0.838	0.688
**History of CVD (%)**	27.27	10.53	19.05	0	0.065	0.349
**Age (year)**	47.55 ± 12.19	55.89 ± 11.15	49.71 ± 14.81	48.95 ± 10.23	0.127	0.080
**BMI (kg/m2)**	23.23 (20.18,24.49)	22.64 (22.07,25.78)	23.18(18.74,25.10)	20.42(22.41,23.94)	0.070	0.087
**BUN (mmol/L)**	26.18 ± 9.32	17.70 ± 5.95^*^	24.81 ± 6.33&	5.75 ± 1.99	<0.001	0.001
**Cre (umol/L)**	897.00 ± 336.63	1019.63 ± 231.73	1114.33 ± 277.21^#^	68.11 ± 14.07	<0.001	0.033
**eGFR (ml/min/1.73m2)**	5.49 (3.37,7.51)	3.72 (3.21,5.09)	3.61 (3.02,4.45) ^#^	93.76 (91.33,114.10)	<0.001	0.035
**UA (umol/L)**	485.00 (429.50,573.50)	428.00 (373.00,496.00)	512.00 (415.50,577.50)	352.00 (292.50,357.50)	<0.001	0.049
**AST (mmol/L)**	16.00 (11.50,18.00)	18.00 (14.00,24.00)	13.00 (10.00,17.00)	21.00(15.00,24.25)	0.044	0.111
**ALT (mmol/L)**	12.00 (8.00,22.50)	13.00(11.00,19.00)	9.00 (7.50,15.00)	18.00 (12.25,23.50)	0.156	0.207
**ALB (g/L)**	36.28 ± 4.02	32.92 ± 3.80^*^	40.11 ± 3.17^#&^	43.33 ± 2.25	<0.001	<0.001
**TC (mmol/L)**	4.05 (3.46,5.13)	3.99 (3.56,4.54)	3.96 (3.06,4.47)	4.64 (4.47,5.20)	0.112	0.354
**TG (mmol/L)**	1.17 (0.91,1.65)	1.08 (0.67,2.18)	1.08 (0.78,1.61)	1.03 (0.67,2.13)	0.957	0.871
**HDL (mmol/L)**	1.10 ± 0.34	1.01 ± 0.26	1.04 ± 0.24	1.12 (1.08,1.42)	0.504	0.511
**LDL (mmol/L)**	2.65 ± 0.87	2.43 ± 0.87	2.32 ± 0.75	3.07 (2.36,3.25)	0.287	0.343
**HB (g/L)**	78.00 (67.00,93.00)	94.00 (84.00,110.00)	108.00 (100.50,114.00) ^#^	137.00 (106.75,153.5)	<0.001	<0.001
**WBC (10^9/L)**	6.15 ± 1.95	6.20 ± 1.83	5.74 ± 1.74	5.64 ± 0.91	0.812	0.672
**Neutrophils (%)**	65.98 ± 8.18	64.50 ± 9.50	60.94 ± 10.34	53.80 ± 3.21	0.060	0.150

ND group, Non-dialysis ESRD patients; PD group, Peritoneal dialysis ESRD patients; HD group, Hemodialysis ESRD patients; CTL group, healthy controls. Normally distributed data were presented as mean (standard deviation), Non-normally distributed data were presented as median (interquartile range). BMI, body mass index; BUN, blood urea nitrogen; Cre, creatinine; eGFR: estimated glomerular filtration rate; UA, uric acid; AST, aspartate transaminase; ALT, alanine amiotransferase; ALB, albumin; TC, total cholesterol; TG, triglycerides; LDL, low-density lipoprotein; HDL, high-density lipoprotein; HB, hemoglobin; WBC, white blood cell count.P1 value was obtained from the comparison were performed among the four groups by ANOVA with Bonferroni and Kruskal-Wallis, as appropriate. P2 value was derived from the comparison between ND, PD and HD groups. The symbol “*” indicated the significant difference between ND Group and PD Group; The symbol “#” indicated the significant difference between ND Group and HD Group; The symbol “&” indicated the significant difference between PD Group and HD Group (P<0.05).

**Table 2:** The clinical characteristics of ESRD patients.

	ND group	PD group	HD group	*P*-value
**CRP (mg/ml)**	1.80 (0.95,3.20)	1.56 (0.71,4.80)	1.20 (0.60,2.08)	0.307
**HCY (umol/L)**	27.83 (19.29,39.07)	18.99 (17.23,26.11)^*^	30.03 (21.76,34.82)	0.019
**TIBC (umol/L)**	37.10 (33.17,41.95)	33.90 (30.80,37.40)	32.70 (28.70,35.90)^#^	0.021
**Serum ferritin(ng/ml)**	115.64 (68.76,266.56)	291.60 (153.75,401.29)	414.62 (243.14,573.76)^#^	<0.001
**PTH (pg/ml)**	336.65 (134.38,578.53)	240.47 (52.43,661.04)	509.22 (359.28,1230.62)^&^	0.028
**Serum potassium (mmol/l)**	4.12 (3.79,4.69)	3.89(3.36,4.38)	4.93 (4.12,5.42)^#&^	0.001
**Serum sodium (mmol/l)**	141.00 (137.50,143.00)	141.00 (139.00,143.00)	140.00 (138.00,142.00)	0.522
**Serum chlorine (mmol/l)**	101.80 (99.35,105.30)	98.00 (96.90,100.20)	95.20 (92.90,98.60)^#^	<0.001
**Serum calcium (mmol/l)**	2.12 ± 0.27	2.29 ± 0.27	2.25 ± 0.26	0.061
**Serum phosphate (mmol/l)**	1.73 ± 0.42	1.45 ± 0.45	1.77 ± 0.49	0.055
**Serum HCO3-(mmol/l)**	19.28 ± 3.58	25.38 ± 2.90*	21.29 ± 3.17&	<0.001
**Serum iron (umol/L)**	11.03 ± 4.77	12.47 ± 4.98	11.00 ± 4.58	0.523
**Serum zinc (umol/L)**	9.10 (7.53,9.84)	8.47 (7.36,8.90)	10.20 (8.98,11.69)^#&^	0.002
**Serum magnesium (mmol/l)**	0.95 ± 0.19	0.83 ± 0.14^*^	1.10 ± 0.16^#&^	<0.001
**Serum copper (umol/L)**	13.20(11.70,15.60)	13.50(11.81,15.30)	14.00(12.55,16.25)	0.637
**Dialysis vintage (years)**		3.00 (2.00,3.00)	3.00 (2.00,5.00)	0.347
**Kt/v**		1.80 (1.52,2.06)	1.59 (1.23,1.80)	0.041

CRP, C-reactive protein; HCY, homocysteine; TIBC, total iron-binding capacity; PTH, parathyroid hormone. P-value was based on ANOVA with Bonferroni and Kruskal-Wallis tests, which were used to compare the clinical characteristics among the three groups. Mann-Whitney U test was carried out to determine differences about dialysis vintage and Kt/v between PD and HD group. The symbol “*” indicated the significant difference between ND Group and PD Group; The symbol “#” indicated the significant difference between ND Group and HD Group; The symbol “&” indicated the significant difference between PD Group and HD Group (P<0.05).

### Bacterial Microbiota Composition and Diversity Analysis

**Figure 1:**
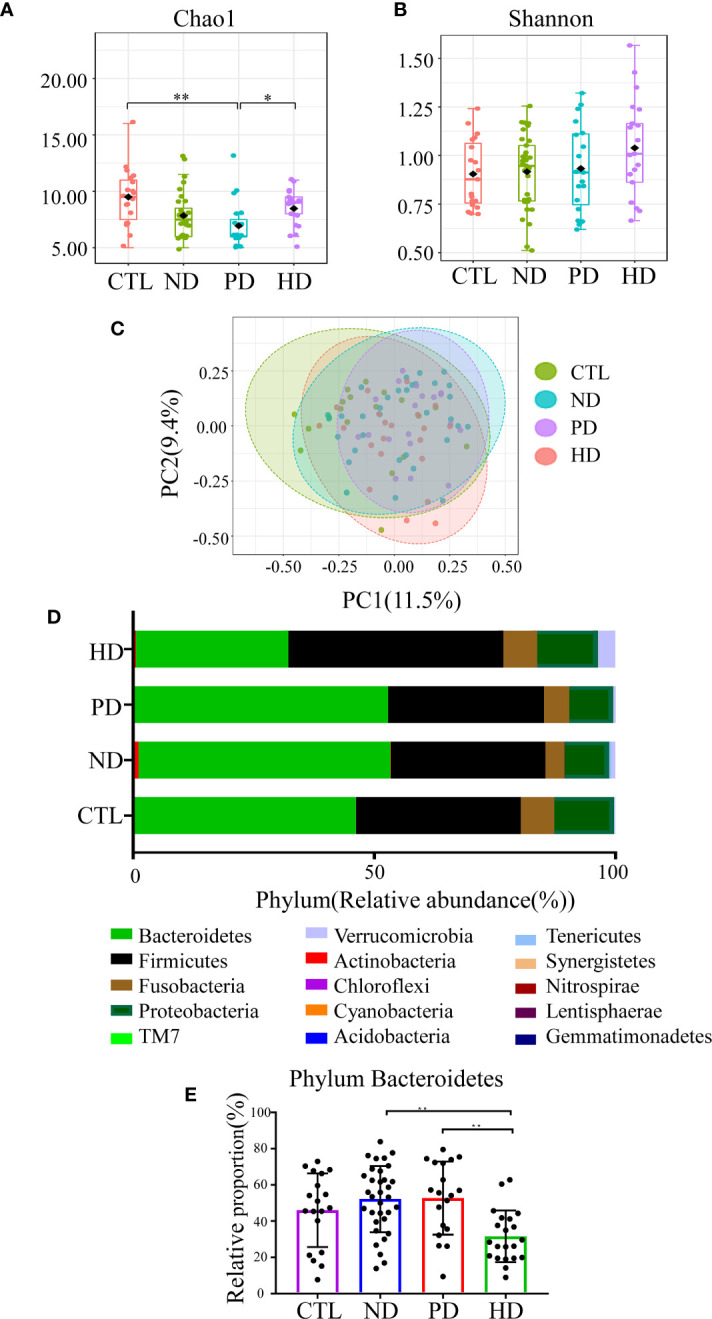
Gut microbiota in ESRD patients and the control group. Alpha diversity was assessed by Chao1 **(A)** (*P*=0.001) and Shannon **(B)** (*P*=0.334), which was compared by Kruskal-Wallis test. **(C)** Visualization of the PCoA based on the Bray-Curtis dissimilarity showed a separation of the microbiota among the four groups. PC1 scores explained 11.5% and PC2 explained 9.4% of total variations. (PERMANOVA) F-value:2.2567; R-squared: 0.071438, *P* < 0.001; (ANOSIM) R: 0.12456; *P* < 0.001. R-squared represents the degree of variation between groups that was explained. The credibility of this test is high (*P* < 0.05). The closer the R value in ANOSIM is to 1, the larger the difference between the groups compared to the difference within the groups (*P* < 0.05). **(D)** The composition of gut microbiota at the phylum level in the CTL, ND, PD, and HD groups. **(E)** Representation of the relative abundances of *Bacteroidetes* among the four groups at the phylum level. *P<0.05; **P<0.01.

Alpha diversity shows species richness and/or evenness within a sample. Chao1 estimates the richness of taxa, and the larger Chao1 index, the more species in the sample. Shannon index takes both species richness and evenness into account, and the larger the Shannon index, the higher the community diversity. Chao1 index was lower in ESRD patients with or without dialysis when compared with the healthy controls (*P* = 0.003), while Shannon index had no difference between groups **(** **Supplementary Figures 1A, B** **)**. There is no statistical difference between ND group and dialysis group in Chao1 and Shannon index **(** **Supplementary Figures 1C, D** **)**. In a detailed analysis, we found Chao1 index in PD patients was significantly lower than that in the control group (*P*=0.001) and HD patients (*P*=0.034) **(** **Figure 1A** **)**. Shannon index showed no significant difference among the four groups **(** **Figure 1B** **)**. Beta diversity was used to assess differences in community composition between groups. Close groups were more similar in terms of microbial community distribution. PCoA analysis showed that the gut microbiota of ESRD patients, whether on dialysis or not, were distinct from healthy controls, in terms of the first two principal component scores 11.5% and 9.4%, respectively. When compared with pre-dialysis patients, HD patients showed significant changes; (PERMANOVA: F-value = 2.2567; R-squared = 0.071438, *P* < 0.001) (**Figure 1C**). In a pairwise comparative analysis, the results of beta diversity between CTL and ESRD (with or without dialysis patients); ND and dialysis patients; CTL and ND group; CTL and PD group; CTL and HD group; ND and HD group; PD and HD group all showed that the difference between groups was greater than that within groups and the difference between groups was significant (*P* < 0.05). (**Supplementary Figures 1E, F** and **Supplementary Figure 2**). For phylum classification, our data showed different gut microbiota composition in each group (**Figure 1D**). Bacterial communities at phyla levels, with relative abundance > 0.1%, and significant differences of *P* < 0.05 were identified; HD patients had the lowest abundance of *Bacteroidetes* (**Figure 1E**).

### Differences in Gut Microbiota Between Pre-Dialysis and Dialysis ESRD Patients

**Figure 2:**
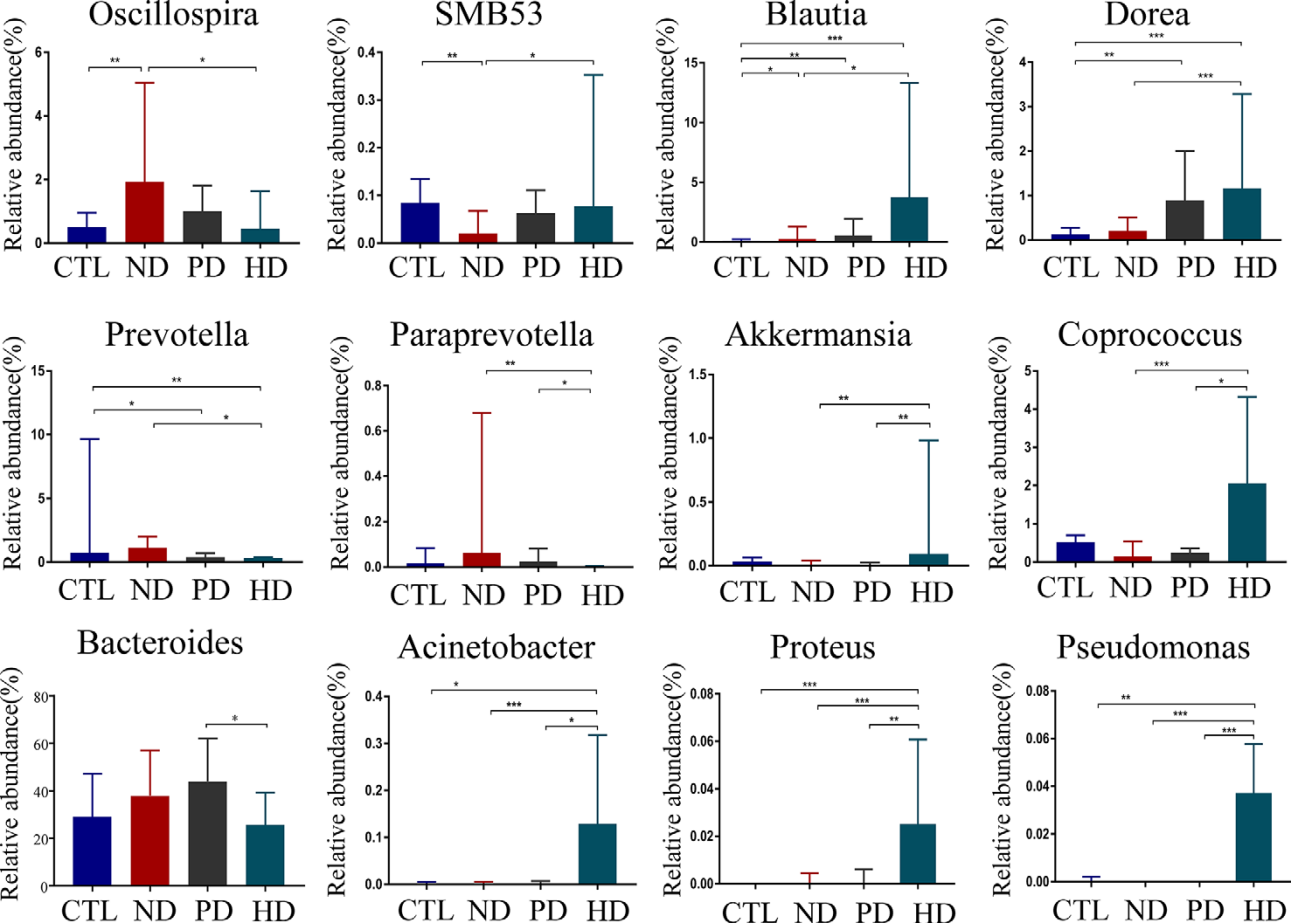
Compositional alterations of gut microbiota at genus level caused by dialysis. There were 12 different genera of bacteria with statistical significance(*P*<0.05) in pre-dialysis and dialysis ESRD patients and healthy controls. The proportion of genus Bacteroides was presented as mean ± SD. Other different bacteria were shown as the median (IQR) in the bar graph. *P<0.05, **P<0.01, ***P<0.001.

*Genus* with statistical differences between CTL and ESRD group with or without dialysis included *Blautia, Clostridium, Dialister, Dorea, Faecalibacterium, Lachnospira, Megamonas, Megasphaera, Oscillospira, Prevotella, Roseburia, Ruminococcus, SMB53, Streptococcus, Veillonella, Clostridium_A* and *Ruminococcus_A* (P < 0.05) **(** **Supplementary Table 1** **)**. To explore gut microbial changes after PD and HD, gut microbiota composition at the genus level were analyzed. The genus included *Blautia, Clostridium, Coprococcus, Dorea, Oscillospira, Parabacteroides, Paraprevotella, Prevotella, SMB53* and *Ruminococcus A* had statistical differences between pre-dialysis and dialysis ESRD patients (P < 0.05) **(** **Supplementary Table 1** **)**. In a detailed analysis, amongst the four groups, 12 genera with statistically significant differences, were related to dialysis (P < 0.05) (**Figure 2**). When compared with healthy controls, pre-dialysis ESRD patients had a significantly higher relative abundance of *Oscillospira* and *Blautia*, and a lower relative abundance of *SMB53*. Both PD and HD patients exhibited increased *Blautia* and *Dorea*, and decreased *Prevotella*. When compared with pre-dialysis patients, the HD group had a lower relative abundance of *Prevotella* and *Paraprevotella*. We also observed a significantly increased abundance of *Akkermansia, Coprococcus, Acinetobacter, Proteus* and *Pseudomonas* in HD patients. Furthermore, HD reversed abnormal changes in these florae in pre-dialysis patients, including increased *Oscillospira*, and decreased *SMB53*. We could not find any bacterial differences between PD and pre-dialysis patients at the genus level. When compared with PD patients, those with HD had significantly lower *Bacteroides*.

### Correlations Between Clinical Markers and Microbiota

**Figure 3:**
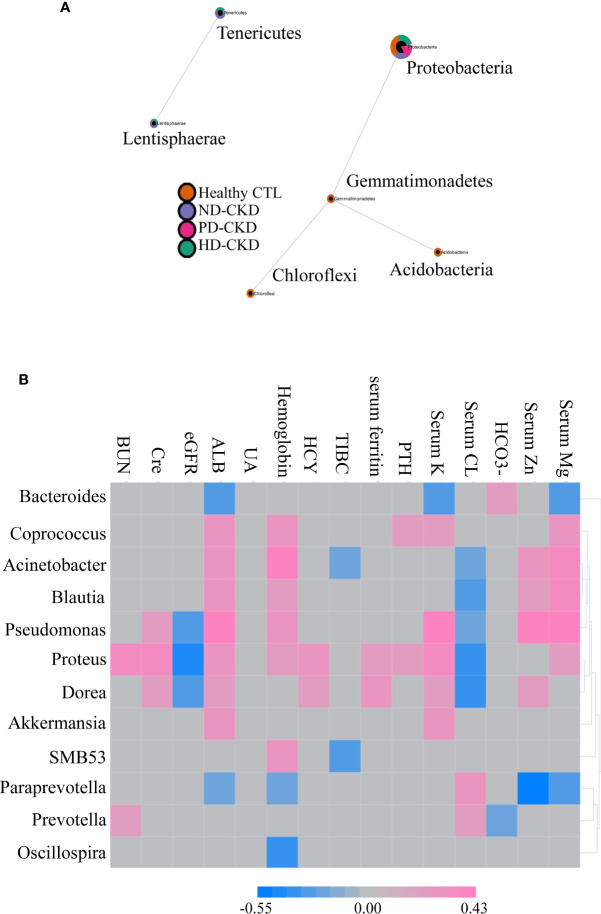
Correlation between gut microbiota composition and clinical parameters. **(A)** Related network graph generated using SparCC algorithm. The image was the correlation network, whose nodes represented taxa at the phylum level, and whose edges represented correlations between the taxa pairs. The nodes were colored according to the relative content between different groups of phylum classification. **(B)** Correlations between different gut microbiota and clinical indicators were reported in the heatmap in the ESRD patients. The grey grid indicated that the correlation was not statistically significant (*P*>0.05). The red grid showed a positive correlation, and the blue grid showed a negative correlation.

The sparCC correlation network identifies potential interactions between bacteria that represent a variety of interrelationships, including symbiotic, parasitic and even competitive relationships. Revealing such interactions may have important implications for microbial communities, and ultimately contribute to microbiome function. Our result revealed that *Gemmatimonadetes* were associated with *Proteobacteria*, *Acidobacteria* and *Chloroflexi* at the phylum level. Similarly, *Lentisphaerae* were correlated with *Tenericutes* (**Figure 3A**). Several significant correlations between the relative abundance of gut microbiota and blood biochemical indicators were noted. *Bacteroides* were negatively associated with serum albumin, potassium, and magnesium levels, but positively associated with serum HCO3-. *Akkermansia* and *SMB53* were positively associated with hemoglobin and negatively correlated with TIBC. *Dorea* was positively correlated with serum creatinine, albumin, HCY, ferritin, potassium, and zinc (**Figure 3B**).

### Gut Microbiota Functional Differences in Study Groups

**Figure 4:**
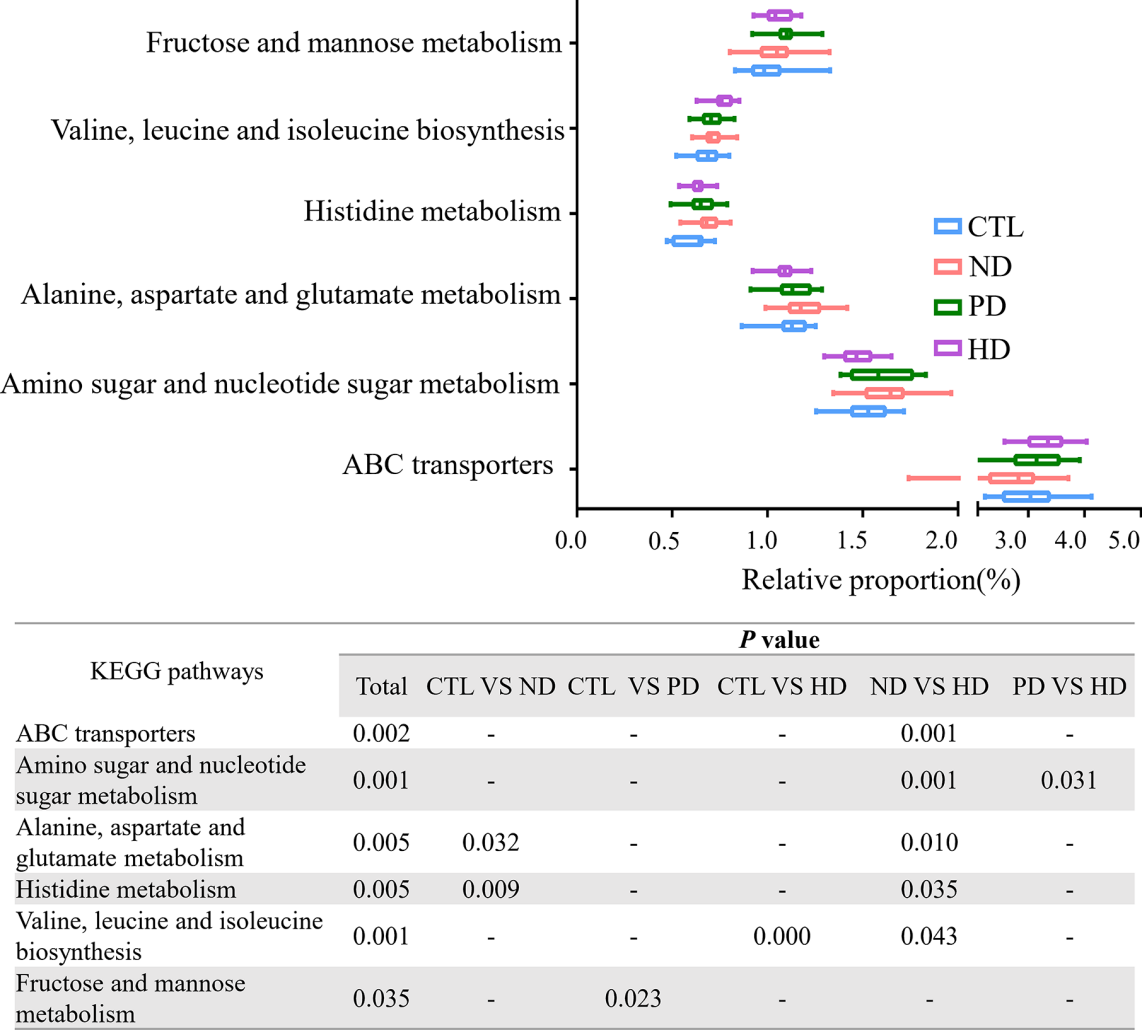
PICRUSt analysis in the KEGG pathways based on 16S sequencing data. Functional predictions for the fecal microbiome among four groups. Pathways with significant differences( *P*<0.05)were screened out. Whiskers: Min to Max. The two ends represented the maximum and minimum values. The middle of the rectangle was the median and the ends of the rectangle were the quartiles. Statistical results of pair comparison of KEGG signaling pathways are shown in the table below. “-” means there was no statistical difference between the two groups.

We used the PICRUSt website to infer the functional content of microbiota based on microbiota annotation and OTU abundance information. We identified six significant signaling pathways by eliminating pathways with too low a content, and those unrelated to ESRD (**Figure 4**). When compared with pre-dialysis patients, HD patients showed enhanced ABC transporters and valine, leucine and isoleucine biosynthesis, but weakened amino sugar and nucleotide sugar metabolism. Alanine, aspartate, glutamate and histidine metabolic pathways were abnormally enhanced in patients before dialysis, while HD could repair this change. When compared with healthy subjects, PD patients enhanced fructose and mannose signaling pathway metabolism.

### Events During Follow-Up

**Figure 5:**
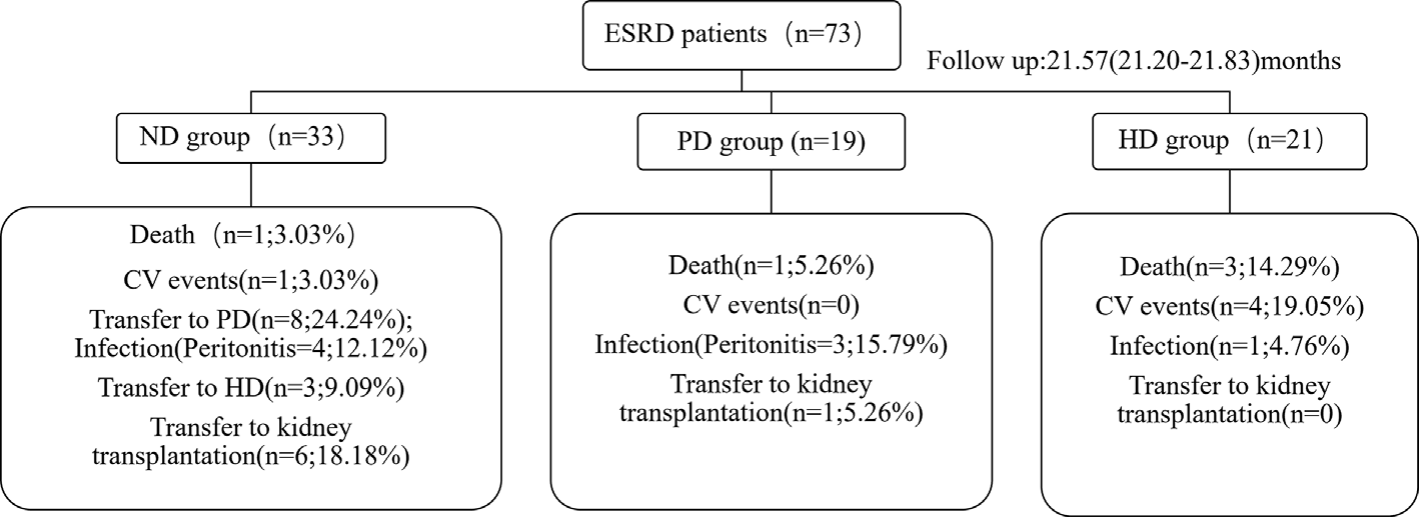
At the end of follow-up, the clinical outcomes of ESRD patients.

**Figure 6:**
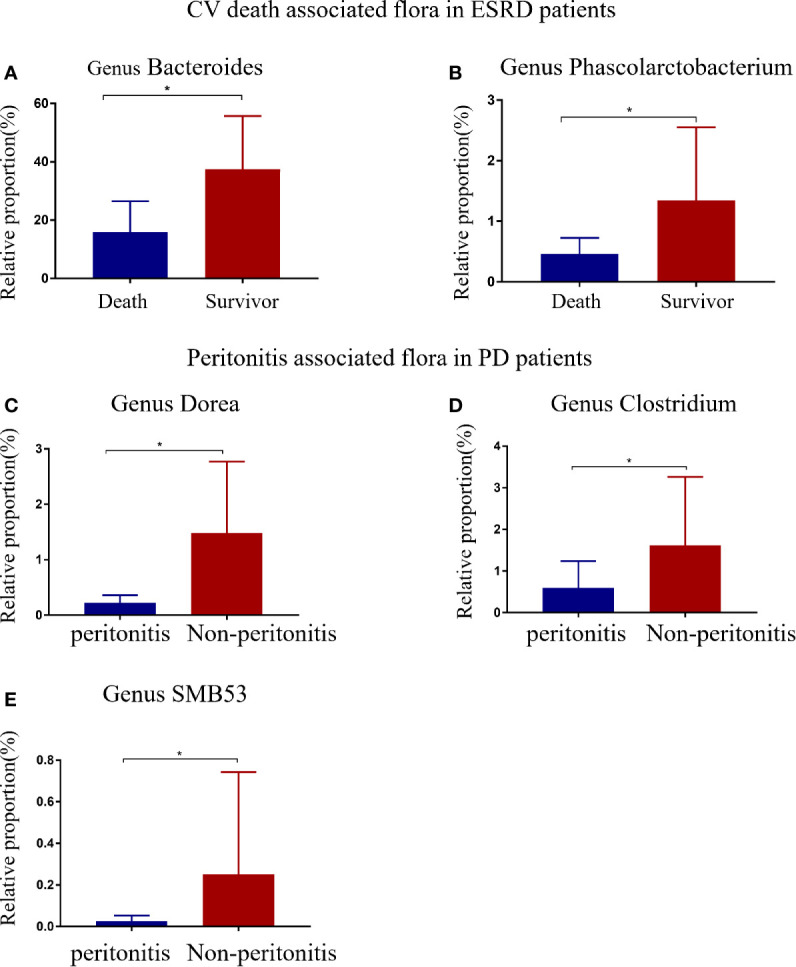
ESRD patients with different clinical outcomes had different gut flora at the genus level. Differential gut microbiota between CV deaths and survivors in ESRD patients **(A, B)**. Compared with PD patients, peritonitis patients showed a decrease in three bacterial genera **(C–E)**. The data is represented by a bar graph with a mean ± SD or median (IQR). The asterisk indicates that there is a significant difference between the two groups (*P*<0.05).

Based on CV disease history and cardiac ultrasound data when collecting patient stool specimens, we observed no statistically significant differences in cardiac function amongst the three patient groups (**Supplementary Table 2**). The median follow-up time for ESRD patients was 25.23 months (IQR: 23.32–29.55 months). Five deaths were recorded, and were due to CV events. Seven patients had a history of peritonitis, including three PD patients, and four pre-dialysis patients who switched to PD (**Figure 5**). The proportions of *Bacteroides* and *Phascolarctobacterium* in the patients with CV death were lower than ESRD survivors (**Figures 6A, B** **)**. When compared with PD patients without peritonitis, those who had peritonitis exhibited a decreased relative abundance of *Dorea*, *Clostridium* and *SMB53* (**Figures 6C–E**).

## Discussion

Our study specifically compared pre-dialysis ESRD patients with PD and HD patients and clarified the role of dialysis in gut microbiota, using high-throughput sequencing. The composition and function of gut microbiota in pre-dialysis and dialysis ESRD patients were altered. These alterations were pronounced in HD patients, who restored the relative abundance of beneficial bacteria, and induced some potential pathogenic bacteria. We observed no bacterial differences between PD and pre-dialysis patients at the phylum and genus levels. Alterations in gut microbiota were related to some clinical parameters about nutritional and electrolyte status. Our PICRUSt data suggested that dialysis triggered gut microbiota alterations in signal transduction and metabolic pathways. To the best of our knowledge, this is the first follow-up study to explore the effects of dialysis treatment on the gut microbiota in patients with ESRD, and to clarify the impact of gut microbiota on clinical outcomes.

Our study observed that the change in gut microbiota in patients with ESRD, which was consistent with many literatures (Wang et al., 2019; Hu et al., 2020b). Besides, gut microbiota was correlated with the clinical indicators of patients, suggesting disease complications and gut microflora dysbiosis may influence each other. CKD associated factors may play a role in the promotion of gut microbiota imbalance, such as increasing intestinal uremic toxin availability, metabolic acidosis, intestinal wall edema, and reducing colonic transit and digestive capacity (Vaziri, 2012; Vaziri et al., 2013). Pharmacological therapies (e.g., antibiotics and iron delivery) may also exert an influence in gut microflora dysbiosis (Sampaio-Maia et al., 2016). Furthermore, we found that the beta diversity and the composition of 11 genera of HD patients were altered when compared to non-dialysis ESRD patients. HD therapy had a significant impact on the gut microbiota of ESRD patients. A similar conclusion from Haidong He et al. reported that, compared with non-dialysis CKD patients, HD could improve gut microbiota disorders, including increasing *Bifidobacteria* and *Lactobacillus acidophilus*, and reducing *Escherichia coli* and *Enterococcus faecalis*. The reason that may partly explain the discrepancies of results between studies is the differences in dialysis adequacy of the study subjects, genetic history, diet and lifestyle. HD repaired intestinal microenvironment homeostasis by removing toxic and harmful substances from ESRD patients. Components of these uremia toxins, e.g., protein-binding compounds such as indolyl sulfate, p-cresol sulfate and homocysteine can be difficult to remove by routine dialysis (Velasquez et al., 2018). Toxin accumulation, as well as dialysis catheter intervention and strict dietary restrictions, could damage intestinal microenvironments, promoting pathogenic bacterial growth, and inhibiting the growth of beneficial bacteria. In addition, no significant effect of PD on the gut microbiota of ESRD patients was found in our study. By contrast, Hu et al. compared the gut microbiota of PD, HD, non-dialysis CKD and healthy control group, and reported that PD reduced microbial diversity, decreased probiotic butyrate production microbiota, and increased urease -, indole - and p-cresol formation microbiota (Hu J, et al., 2020). The main reason that may explain the difference of results between our study and the study by Hu et al. is likely that the differences in the inflammatory state and age of the subjects, which are known to have important influences on the gut microbiota (Borges et al., 2016; Takagi et al., 2019). In the study of Hu et al., serum CRP in PD patients was significantly higher than that in HD group and non-dialysis group. However, the inflammatory status of the subjects in our study did not differ significantly among groups. In the literature, the disparate findings of both dialysis modalities may be explained as follows. Firstly, the reduction of visceral blood flow under the compensatory mechanism of HD maintaining hemodynamic stability during ultrafiltration may cause intestinal hypo-perfusion, which disrupts intestinal barriers and increases the risk for bacterial translocation (Ribitsch et al., 2015). Secondly, gastrointestinal micro-bleeds induced by systemic anticoagulation therapies during HD treatments, in combination with uremic platelet dysfunction, may impair gut epithelial barrier structures and functions (Vaziri et al., 2016). Lastly, it has been shown that diet plays a role in regulating the composition and metabolic activity of human gut microbiota (Conlon and Bird, 2014). HD patients have more stringent dietary restrictions than PD patients.

*Bacteroides* and *Phascolarctobacterium* were reduced in patients who died from CV events. This observation suggested that *Bacteroides* and *Phascolarctobacterium* may reduce mortality in ESRD patients. A similar finding also observed that growth of the succinate-consuming bacteria *Phascolarctobacterium* spp., prevented *Clostridioides difficile* infection, and therefore, reduced mortality in infected animals (Nagao-Kitamoto et al., 2020). *Bacteroides* maintains a complex relationship with the host; it includes a beneficial role as a commensal organism, and is potentially harmful in human disease (Wexler, 2007). Yoshida et al. showed that the abundance of genus *Bacteroides* was lower in patients with coronary artery disease and further revealed that *Bacteroides vulgatus* and *Bacteroides dorei* inhibited atherosclerosis by reducing the production of intestinal microbial lipopolysaccharides (Yoshida et al., 2018). Besides, the intestinal *Bacteroides* strains directly modulated gut function (Freitas et al., 2003). Our data showed HD patients had significantly lower *Bacteroides* and *Bacteroides* were negatively associated with serum albumin, potassium, and magnesium. A possible mechanism for this observation could be that subnormal *Bacteroides* levels caused by HD disruption of intestinal functional homeostasis have side effects on the ion and nutrient absorption in the gut (Wexler, 2007), thus, increasing CV risk and death. However, *Bacteroides fragilis*, major disease-causing *Bacteroides* species, can contribute to anaerobic bacteremia and sepsis by producing toxins and activating related proteases (Choi et al., 2016). There are many known and unknown subclasses of *Bacteroides*, and these species may have opposite effects on the human. Therefore, more researches are needed to clarify the role of Bacteroides. The role of *Dorea* and *SMB53* in the human body are still poorly understood. Studies have reported that *SMB53* is significantly higher in females, with hard consistency stools (Takagi et al., 2019), and may reduce chronic diarrhea in healthy volunteers with loose stools (Hatanaka et al., 2018). We observed that the relative abundance of *Dorea*, *Clostridium*, and *SMB53* was lower in PD patients with peritonitis, suggesting they may have anti-inflammatory roles. The *Clostridium* genus clusters, IV and XIVa have also been implicated in the maintenance of mucosal homeostasis and may promote CD4+ T regulatory cell accumulation to prevent colitis (Atarashi et al., 2011), supporting our findings.

*Oscillospira* was negatively associated with hemoglobin; previous studies have reported that this strain was abnormally increased in patients with high-bile levels, gallstone formation (Keren et al., 2015) and atopic dermatitis (Reddel et al., 2019). These observations suggested HD could restore abnormal alterations in potentially beneficial bacteria in ESRD patients. Everard et al. demonstrated that *Akkermansia* played a key role in maintaining mucin layer integrity and reducing inflammation (Everard et al., 2013). *Coprococcus* produced short chain fatty acids (SCFAs), especially butyric acid, which exerted multiple critical roles in maintaining human health (Peng et al., 2009; den Besten et al., 2013; Keku et al., 2015). These genera were significantly increased in HD patients and have been shown to exert some health benefits to the host. It was previously shown that *Blautia* may induce protective effects on the body, including decreased obesity (Kimura et al., 2013), and produced butyric acid and acetic acid (Liu et al., 2015). *Blautia* and *Dorea* abundance was increased in PD and HD patients when compared with healthy controls, and were higher in HD than pre-dialysis patients. These data indicated that gut microbiome composition, affected by dialysis, had a positive impact on ESRD patients.

Gut microbiota alterations caused by HD may also induce adverse effects. HD reduced the relative abundance of *Bacteroides* in patients and contributed to adverse patient prognoses. Members of *Acinetobacter* and *Proteus* genera have recently emerged as opportunistic human pathogens, causing severe infections (Harding et al., 2016; Wong et al., 2017). *Proteus* species express many virulence factors potentially related to pathogenicity in the gastrointestinal tract, including motility, adherence, the production of urease, hemolysins and IgA proteases, and acquisition of antibiotic resistance (Hamilton et al., 2018). The *Pseudomonas* genus includes the major human pathogen, *Pseudomonas aeruginosa* (De Smet et al., 2017), which is significantly increased in patients with HD, increasing the risk of infection.

Data on alterations in metabolism-related signaling pathways were derived by PICRUSt analysis, delineating dialysis effects on gut microbiota. ABC transporters have diverse functions; they introduce essential nutrients and export substrates outside the cell (Beis, 2015), they influence the pharmacokinetics and pharmacodynamics of many drugs (Aszalos, 2008), and disease related toxins have been shown to modulate transporter proteins (Aszalos, 2008). HD could effectively eliminate toxin accumulation in ESRD patients, and repair transporters. In our study, genes related to basic metabolisms, such as amino acids, sugars and nucleotides, and alanine, aspartate, glutamate, histidine, valine, leucine and isoleucine biosynthesis were up-regulated in ESRD patients, suggesting impaired protein degradation and absorption mechanisms. High levels of undigested amino acids can accumulate in the colon, increasing nitrogen availability, potentially favoring proteolytic bacteria and forming toxic metabolites (Liu et al., 2018). HD partially improved amino acid metabolic disorders in the colon, however, valine, leucine and isoleucine biosynthesis were pronounced in HD patients, in agreement with data from ultra-performance liquid chromatography/mass spectrometry analyses of fecal metabolic profiles in HD patients (Liu et al., 2018). The overrepresented fructose and mannose metabolism in PD patients may be related to high glucose levels in peritoneal dialysate.

Our study had several shortcomings. Firstly, although our data revealed that gut microbiota was altered at different taxonomic levels, the sample size was relatively limited, therefore, further studies with larger sample numbers are required. Secondly, all subjects were recruited from one center, which shared a limited geographical area and may have been biased in terms of participant diet. Our study did not assess patient diets and drug intake, therefore, the influence of these factors on study data cannot be ruled out. Thirdly, the fecal samples from patients were not collected multiple times, which could better observe the dynamic changes of gut microbiota. Further studies are warranted to clarify the role of PD and HD therapy on the fecal microbiome of ESRD patients.

## Conclusions

Our study suggests that gut microbiota in pre-dialysis, PD, HD patients not only differs at taxonomic levels, but also at functional levels, involving different metabolic pathways. When compared with pre-dialysis patients, changes in the gut microbiota of HD patients were more significant than PD patients. Our data shows that the intestinal microbiota has an interactive relationship with clinical outcomes of ESRD patients. Therefore, the identification of alterations in gut microbiota during dialysis will undoubtedly assist physicians in administering preventative and timely interventions to avoid mortality risks in ESRD patients.

## Data Availability Statement

The datasets presented in this study can be found in online repositories. The names of the repository/repositories and accession number(s) can be found below: NCBI, accession number PRJNA646021.

## Ethics Statement

The studies involving human participants were reviewed and approved by the ethics review board of The Third Affiliated Hospital of Sun Yat-sen University. The patients/participants provided their written informed consent to participate in this study.

## Author Contributions

Conceptualization: DL, ZL, JZ, and HP. Data curation: JW and JS. Investigation: JW and YL. Methodology: DL, WZ, ZL, and HL. Formal analysis and Writing—original draft: DL and WZ. Project administration and writing—review and editing: JZ and HP. All authors contributed to the article and approved the submitted version.

## Funding

This research was funded by “The Project of Cultivating Young Teachers in Sun Yat-sen University, grant number 17ykzd22”; “Guangzhou Science and Technology Project, grant number 201807010037”; “Guangdong Basic and Applied Basic Research Foundation, grant number 2020A1515011287”; “The Science and Technology Planning Project Foundation of Guangdong Province, grant number 2016A020215071”; and “The Medical Scientific Research Foundation of Guangdong, grant number A2016227”.

## Conflict of Interest

The authors declare that the research was conducted in the absence of any commercial or financial relationships that could be construed as a potential conflict of interest.
