# Supplementary material for: The Effects of Hemodialysis and Peritoneal Dialysis on the Gut Microbiota of End-Stage Renal Disease Patients, and the Relationship Between Gut Microbiota and Patient Prognoses
Source: Front Cell Infect Microbiol. 2021 Mar 23;11:579386. doi: 10.3389/fcimb.2021.579386 (PMC8021868; doi:10.3389/fcimb.2021.579386)
Supplement: Supplementary file 1 [file DataSheet_1.docx]

Supplementary Material

**Supplementary Figure 1**. The alpha and beta diversity in control and ESRD patients. The chao1(A) and Shannon (B) index at the phylum level between CTL and ESRD (with or without dialysis patients) (*P*= 0.003; *P*=0.286 , respectively). The chao1(C) and Shannon (D) index at the phylum level between ND and dialysis patients (*P*= 0.7244; *P*=0.182, respectively). (E) The beta diversity between CTL and ESRD ((with or without dialysis patients), [PERMANOVA] F-value: 2.845; R-squared: 0.030643; *P* < 0.001; [ANOSIM] R: 0.16243; *P* < 0.011. (F) The beta diversity between ND and dialysis patients [PERMANOVA] F-value: 1.931; R-squared: 0.026477; *P*< 0.006；[ANOSIM] R: 0.05308; *P* < 0.015.

**Supplementary Figure 2.** The results of pairwise comparison from the PCoA result. (A) Statistical results were compared between CTL group and ND group. [PERMANOVA] F-value: 2.6161; R-squared: 0.04972; *P* < 0.001; [ANOSIM] R: 0.15976; *P* < 0.006. (B) Statistical results were compared between CTL group and PD group. [PERMANOVA] F-value: 2.5113; R-squared: 0.06521; *P* < 0.003; [ANOSIM] R: 0.15796; *P* < 0.001.(C) Statistical results were compared between CTL group and HD group. [PERMANOVA] F-value: 2.5909; R-squared: 0.063829; *P* < 0.001; [ANOSIM] R: 0.18329; *P* < 0.001.(D) Statistical results were compared between ND group and PD group. [PERMANOVA] F-value: 1.213; R-squared: 0.023686; *P* < 0.182; [ANOSIM] R: 0.01257; *P* < 0.365.(E) Statistical results were compared between ND group and HD group. [PERMANOVA] F-value: 2.6872; R-squared: 0.049137; *P* < 0.001; [ANOSIM] R: 0.149; *P* < 0.003.(F) Statistical results were compared between ND group and HD group. [PERMANOVA] F-value: 1.9877; R-squared: 0.049707; *P* < 0.005; [ANOSIM] R: 0.10928; *P* < 0.004.

Supplementary Table 1 The different genera between CTL and ESRD group and ND and dialysis group.

| Genus | CTL group | ESRD group | CTL VS ESRD  *P*-value FDR-corrected | ND group | Dialysis group | ND VS Dialysis  *P*-value FDR-corrected |
| --- | --- | --- | --- | --- | --- | --- |
| Akkermansia | 0.032(0.026,0.063) | 0.014(0.002,0.0948) | 0.179 | 0.008(0.003,0.041) | 0.048(0.002,0.157) | 0.248 |
| Bacteroides | 34.306(14.654,43.690) | 34.915(19.103,52.494) | 0.258 | 37.841±19.197 | 34.288±18.326 | 0.569 |
| Bifidobacterium | 0.012(0.003,0.068) | 0.017(0.004,0.100) | 0.698 | 0.009(0.004,0.057) | 0.037(0.003,0.117) | 0.403 |
| Blautia | 0.095(0.046,0.228) | 0.708(0.190,3.807) | ＜0.001 | 0.273(0.111,1.280) | 1.235(0.240,5.550) | 0.027 |
| Clostridium | 0.257(0.108,0.782) | 0.687(0.336,1.318) | 0.016 | 0.391(0.227,0.805) | 0.779(0.427,1.878) | 0.031 |
| Clostridium_A | 0.008(0.002,0.016) | 0.039(0.007,0.123) | 0.043 | 0.034(0.001,0.129) | 0.054(0.020,0.124) | 0.299 |
| Coprococcus | 0.525(0.197,0.703) | 0.263(0.107,1.235) | 0.747 | 0.142(0.069,0.541) | 0.360(0.184,2.365) | 0.021 |
| Dialister | 0.098(0.052,0.374) | 0.008(0.001,0.024) | ＜0.001 | 0.005(0.000,0.023) | 0.010(0.004,0.036) | 0.179 |
| Dorea | 0.126(0.071, 0.274) | 0.504(0.189,1.565) | ＜0.001 | 0.202(0.121,0.508) | 1.161(0.439,2.529) | ＜0.001 |
| Escherichia | 0.564(0.313,9.358) | 1.469(0.287,5.700) | 0.716 | 0.722(0.246,7.049) | 1.955(0.301,5.564) | 0.682 |
| Eubacterium | 0.008(0.004,0.020) | 0.057(0.015,0.228) | ＜0.001 | 0.039(0.009,0.205) | 0.069(0.021,0.280) | 0.381 |
| Faecalibacterium | 4.627(1.407,15.690) | 1.814(0.450,5.117) | 0.014 | 1.661(0.500,4.266) | 1.880(0.421,6.003) | 0.765 |
| Fusobacterium | 0.438(0.259,4.074) | 0.371(0.080,6.489) | 0.502 | 0.150(0.048,2.285) | 0.929(0.112,7.042) | 0.087 |
| Haemophilus | 0.018(0.008,0.131) | 0.010(0.005,0.042) | 0.127 | 0.010(0.005,0.044) | 0.011(0.005,0.042) | 1.000 |
| Klebsiella | 0.190(0.138, 1.028) | 0.094(0.041, 0.541) | 0.064 | 0.085(0.029,0.352) | 0.128(0.055,0.918) | 0.189 |
| Lachnospira | 3.268(0.675,8.124) | 0.475(0.092,1.205) | 0.007 | 0.480(0.042,1.046) | 0.467(0.166,1.750) | 0.244 |
| Megamonas | 0.093(0.064,1.368) | 0.026(0.007,0.096) | 0.004 | 0.014(0.007,0.503) | 0.028(0.007,0.079) | 0.918 |
| Megasphaera | 0.031(0.025,0.069) | 0.000(0.000,0.008) | ＜0.001 | 0.002(0.000,0.009) | 0.000(0.000,0.006) | 0.690 |
| Odoribacter | 0.008(0.003,0.088) | 0.052(0.001,0.140) | 0.054 | 0.109(0.000,0.312) | 0.015(0.002,0.106) | 0.228 |
| Oscillospira | 0.504(0.274,0.954) | 1.307(0.397,2.734) | ＜0.001 | 1.925(0.732,5.043) | 0.590(0.292,1.786) | 0.016 |
| Parabacteroides | 0.262(0.105,1.563) | 1.739(0.716,4.726) | 0.771 | 3.855(1.599,5.518) | 1.373(0.551,2.846) | 0.016 |
| Paraprevotella | 0.016(0.011,0.083) | 0.018(0.002,0.254) | 0.257 | 0.062(0.010,0.680) | 0.006(0.002,0.032) | 0.028 |
| Phascolarctobacterium | 0.273(0.092,2.281) | 1.291(0.302,2.401) | 0.247 | 1.608(0.557,2.978) | 0.903(0.104,2.092) | 0.238 |
| Prevotella | 0.737(0.534,9.653) | 0.415(0.177,1.356) | 0.022 | 1.105(0.286,1.991) | 0.303(0.134,0.558) | 0.025 |
| Roseburia | 2.460(1.021, 4.745) | 0.350(0.138,1.729) | 0.004 | 0.346(0.061,0.628) | 0.369(0.189,2.060) | 0.291 |
| Ruminococcus | 0.207(0.129,0.765) | 0.896(0.262,2.816) | 0.021 | 1.063(0.293,2.167) | 0.757(0.201,3.697) | 0.809 |
| Ruminococcus_A | 0.487(0.349,1.042) | 1.276(0.637,3.730) | 0.009 | 0.923(0.395,2.252) | 2.128(0.931,4.455) | 0.021 |
| SMB53 | 0.084(0.057,0.135) | 0.050(0.009,0.092) | 0.019 | 0.020(0.007,0.067) | 0.074(0.022,0.177) | 0.025 |
| Streptococcus | 0.022(0.004,0.048) | 0.043(0.018,0.176) | 0.014 | 0.041(0.013,0.185) | 0.048(0.020, 0.169) | 0.696 |
| Sutterella | 0.307(0.078,0.998) | 0.164(0.064,1.078) | 0.438 | 0.221(0.070,1.674) | 0.115(0.055,0.364) | 0.326 |
| Veillonella | 0.085(0.028,0.283) | 0.020(0.010,0.052) | 0.016 | 0.016(0.009,0.056) | 0.021(0.010,0.035) | 1.000 |

**Supplementary Table 2** Cardiac ultrasound data in ESRD patients.

|  | ND group | PD group | HD group | *P-*value |
| --- | --- | --- | --- | --- |
| cardiovascular disease（%） | 27.27 | 5.26 | 33.33 | 0.093 |
| cerebrovascular disease （%） | 0 | 15.79 | 4.76 | **0.028** |
| arteriosclerosis（%） | 24.24 | 10.53 | 4.76 | 0.146 |
| aortic root (mm) | 29.36±2.40 | 29.42±1.71 | 27.90±2.61 | **0.050** |
| atrium sinistrum (mm) | 33.00(30.50,36.00) | 33.00(32.00,38.00) | 38.00(30.00,41.00) | 0.258 |
| right ventricular outflow tract (mm) | 20.42±2.74 | 21.21±2.27 | 21.19±2.38 | 0.430 |
| interventricular septum (mm) | 12.00(11.00,12.50) | 12.00(12.00,13.00) | 11.00(10.00,13.00) | 0.259 |
| left ventricular end-diastolic volume (mm) | 48.03±4.74 | 48.32±4.37 | 47.10±5.71 | 0.706 |
| left ventricular posterior wall (mm) | 11.00(10.00,12.00） | 11.00(10.00,11.00） | 11.00(10.00,11.50） | 0.932 |
| the length of right atrium (mm) | 42.00(37.50,46.00） | 43.00(39.00,46.00） | 44.00(40.50.47.50） | 0.194 |
| the width of right atrium (mm) | 33.15±3.62 | 34.00±4.07 | 34.71±4.72 | 0.385 |
| the main pulmonary artery (mm) | 23.00(22.00,25.00） | 24.00(22.00,25.00） | 23.00(20.00,24.00） | 0.135 |
| EF（%） | 67.00(62.00,69.50） | 65.00(61.00,69.00） | 63.00(61.00,70.00） | 0.768 |
| FS（%） | 36.12±6.13 | 35.32±4.22 | 36.05±6.84 | 0.886 |
| E（m/s） | 0.70(0.59,0.95） | 0.80(0.60,0.90） | 0.90(0.73,1.20） | 0.056 |
| A（m/s） | 0.91±0.21 | 0.92±0.21 | 0.86±0.18 | 0.615 |
| E/A | 0.76(0.66,1.00） | 0.75(0.63,1.09） | 0.89(0.75,1.45） | 0.059 |
| Platelet(10^9/L) | 171.00（145.50,231.50） | 222.00（168.00,254.00） | 198.00（150.00,253.00） | 0.495 |
| Pulse(/min) | 83.70±12.85 | 79.95±11.43 | 79.81±10.92 | 0.402 |

EF, ejection fraction; FS, fractional shortening; E, the wave of an early peak diastolic velocity; A, the wave of a second peak during atrial contraction; *P* value was obtained from the comparison between ND, PD and HD groups were performed by ANOVA with Bonferroni and Kruskal-Wallis tests, as appropriate.
