# Supplementary figures and images for: The Effects of Hemodialysis and Peritoneal Dialysis on the Gut Microbiota of End-Stage Renal Disease Patients, and the Relationship Between Gut Microbiota and Patient Prognoses
Source: Front Cell Infect Microbiol. 2021 Mar 23;11:579386. doi: 10.3389/fcimb.2021.579386 (PMC8021868; doi:10.3389/fcimb.2021.579386)

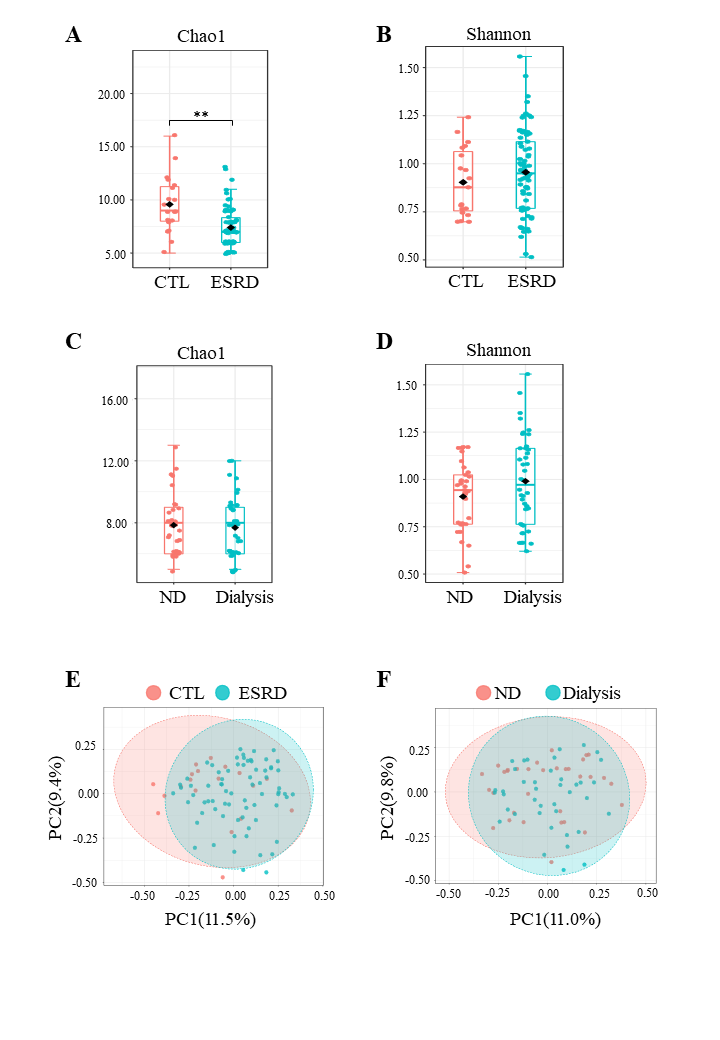

Supplement: Supplementary file 2 [file Image_1.tif]

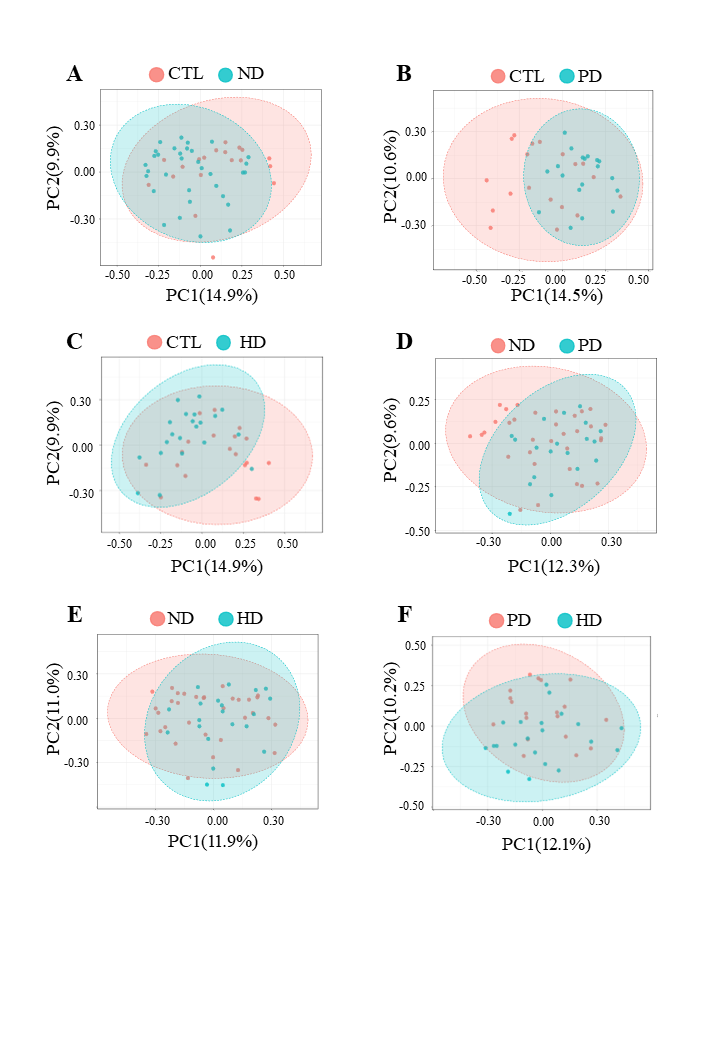

Supplement: Supplementary file 3 [file Image_2.tif]
